# Progestins and the endometrium in patients receiving tamoxifen

**DOI:** 10.1038/sj.bjc.6690473

**Published:** 1999-06


*British Journal of Cancer* (1999) **80**(7), 1114
© 1999 Cancer Research Campaign
Article no. bjoc.1998.0473

Letter to the Editor

### Progestins and the endometrium in patients receiving tamoxifen

### Sir

Since the anti-oestrogen tamoxifen has partial agonist, oestrogen-like effects on the uterus, progestins could theoretically be used – as in hormone replacement therapy (HRT) – to protect the uterus from excessive stimulation. There are, however, very few data on the effects of this association: therefore, we read with interest an article by Powles et al (*Br J Cancer* **78**: 272–275), who used norethisterone in healthy, post-menopausal women receiving tamoxifen in a chemoprevention trial. In this study, 3 months of cyclic progestin therapy failed to reverse endometrial abnormalities identified by transvaginal ultrasound (TVUS).

We observed a similar phenomenon in a series of patients with early breast cancer. Fourteen post-menopausal patients with an intact uterus, who had been on adjuvant tamoxifen (20 mg $day^{-1}$) for a median of 10.5 months (range 1–43) received additional progestin therapy as a treatment of hot flushes. Four patients had megestrol acetate (40 mg $day^{-1}$ p.o.) for 6 consecutive weeks, and ten received three fortnightly intramuscular injections of a depot formulation of 500 mg medroxyprogesterone acetate. Before starting treatment with the progestin, all patients underwent TVUS which showed normal endometrium ($<$ 4 mm thick) in eight patients (57.1%) and endometrial thickening (median 12.5 mm, range 6–20) in six (42.9%). Both megestrol acetate and medroxyprogesterone acetate were well-tolerated, and no patient reported withdrawal bleeding. TVUS measurements were repeated in 12 patients after progestin therapy, showing a thickened endometrium (median 16 mm, range 4–46) in 11 (91.6%). During a median follow-up time of 24 months (range 9–27), endometrial samples were obtained in seven patients (including the two patients who did not repeat TVUS), showing benign tamoxifen-associated abnormalities.

In another report, endometrial biopsies and TVUS measurements were performed in 12 post-menopausal breast cancer patients on tamoxifen who received additional progestins for a median of 5 months (range 1–9) (Cohen et al, 1989). A decidual reaction was observed in 11/12 patients (91.6%), while endometrial thickness measured by TVUS remained abnormal (median thickness 18 mm, range 6–40). Perhaps due to excessive stromal decidualization and oedema after tamoxifen priming, progestins have also been associated with the development of benign megapolyps in breast cancer patients, leading to hysterectomy (Berezowsky et al, 1994).

Taken together, these findings suggest that short-term addition of progestins is not able to reverse or prevent tamoxifen-associated uterine abnormalities detected by TVUS. As in HRT, effective endometrial protection could probably be obtained with a regular, prolonged co-administration of progestins and tamoxifen, starting from the beginning of adjuvant hormone treatment. At present, however, this is not recommended because detrimental effects on disease-free and overall survival of patients cannot be excluded. Thus, the only indication for the addition of progestins in this setting remains the relief of hot flushes (Loprinzi et al, 1994), which can be obtained with a very short (1–2 months) cycle of treatment. Alternative strategies for endometrial protection, such as progestagen-loaded intrauterine devices (Neven and Vergote, 1998), deserve to be investigated.

*G Bertelli, M Venturini, M Bergaglio, C Gustavino and R Rosso*
*National Cancer Institute, Genova, Italy*

*M Valenzano*
*Department of Obstetrics and Gynecology,*
*University of Genova, Italy*

### REFERENCES

Berezowsky J, Chalvardjian A and Murray D (1994) Iatrogenic endometrial megapolyps in women with breast carcinoma. *Obstet Gynecol* **84**: 727–730.

Cohen I, Figer A, Altaras MM, et al (1989) Common endometrial decidual reaction in postmenopausal breast cancer patients treated with tamoxifen and progestogens. *Int J Gynecol Pathol* **8**: 125–131

Loprinzi CL, Michalak JC, Quella SK, et al (1994) Megestrol acetate for the prevention of hot flashes. *N Engl J Med* **331**: 347–352

Neven P and Vergote I (1998) Should tamoxifen users be screened for endometrial lesions? *Lancet* **351**: 155–157

Powles TJ, Bourne TH, Athanasiou S, et al (1998) The effects of norethisterone on endometrial abnormalities identified by transvaginal ultrasound screening of healthy post-menopausal women on tamoxifen or placebo. *Br J Cancer* **78**: 272–275

